# Paclitaxel increases the sensitivity of lung cancer cells to lobaplatin via PI3K/Akt pathway

**DOI:** 10.3892/ol.2021.12480

**Published:** 2021-01-21

**Authors:** Dongjie Ma, Shanqing Li, Yushang Cui, Li Li, Hongsheng Liu, Yeye Chen, Xiaoyun Zhou

Oncol Lett 15: 6211-6216, 2018; DOI: 10.3892/ol.2018.8086

Following the publication of the above paper, it was drawn to the authors’ attention by an interested reader that the scratch-wound assay images featured in Fig. 5A were, broadly speaking, rather similar, even though they were intending to show the results from three separate experiments, and there also appeared to be repeating, identical cellular patterns within the images themselves in this Figure part. The authors replied to the Editorial Office to explain that errors were made during the process of uploading the images, and also to request a retraction of the paper.

The Editor of *Oncology Letters* has concurred that this article should be retracted, and all the authors agreed with the retraction. The Editor apologizes to the readership for any inconvenience caused.

